# The temporal dynamics of suicide ideation and affective, cognitive, social and contextual symptoms during the COVID-19 pandemic: A dynamic time warp analysis

**DOI:** 10.1192/j.eurpsy.2026.10675

**Published:** 2026-07-31

**Authors:** A. J. C. Van Der Slot, C. Boonmann, M. Eikelenboom, M. W. Gijzen, D. De Beurs, E. J. Giltay

**Affiliations:** 1 Psychiatry; 2 Child and Adolescent Psychiatry, Leiden University Medical Center, Leiden; 3 Amsterdam University Medical Center; 4 University of Amsterdam, Amsterdam, Netherlands

## Abstract

**Introduction:**

Suicidal ideation (SI) is a significant global mental health concern, yet the dynamic interplay between SI and other symptoms remains poorly understood. Clarifying which symptoms co-fluctuate with SI over time may help identify early warning signals and actionable intervention targets.

**Objectives:**

This study aimed to determine which affective, cognitive, and interpersonal symptoms align most strongly with SI across weeks and months.

**Methods:**

from three Dutch psychiatric cohorts of individuals with lifetime internalizing disorders spanning 16 waves from April 2020 until February 2022 (COVID-19 pandemic) were analyzed, including only participants with any fluctuation in SI over time. Variables included depressive, happiness, anxiety, loneliness, worry symptoms and COVID-19-specific items. Dynamic Time Warping (DTW) was applied to quantify within-person (dis)similarity between symptom trajectories, and results were subsequently aggregated at group level.

**Results:**

The 307 participants were on average 44.8 years old and 61.6% was female. Their average SI levels increased over the 16 waves. SI was significantly aligned with four depressive symptoms (i.e., sad mood, low self-esteem, low interest, and reduced happiness), two anxiety-related symptoms (e.g., fear of losing control, faintness), feeling abandoned, and overwhelming worrying.

**Image 1:**
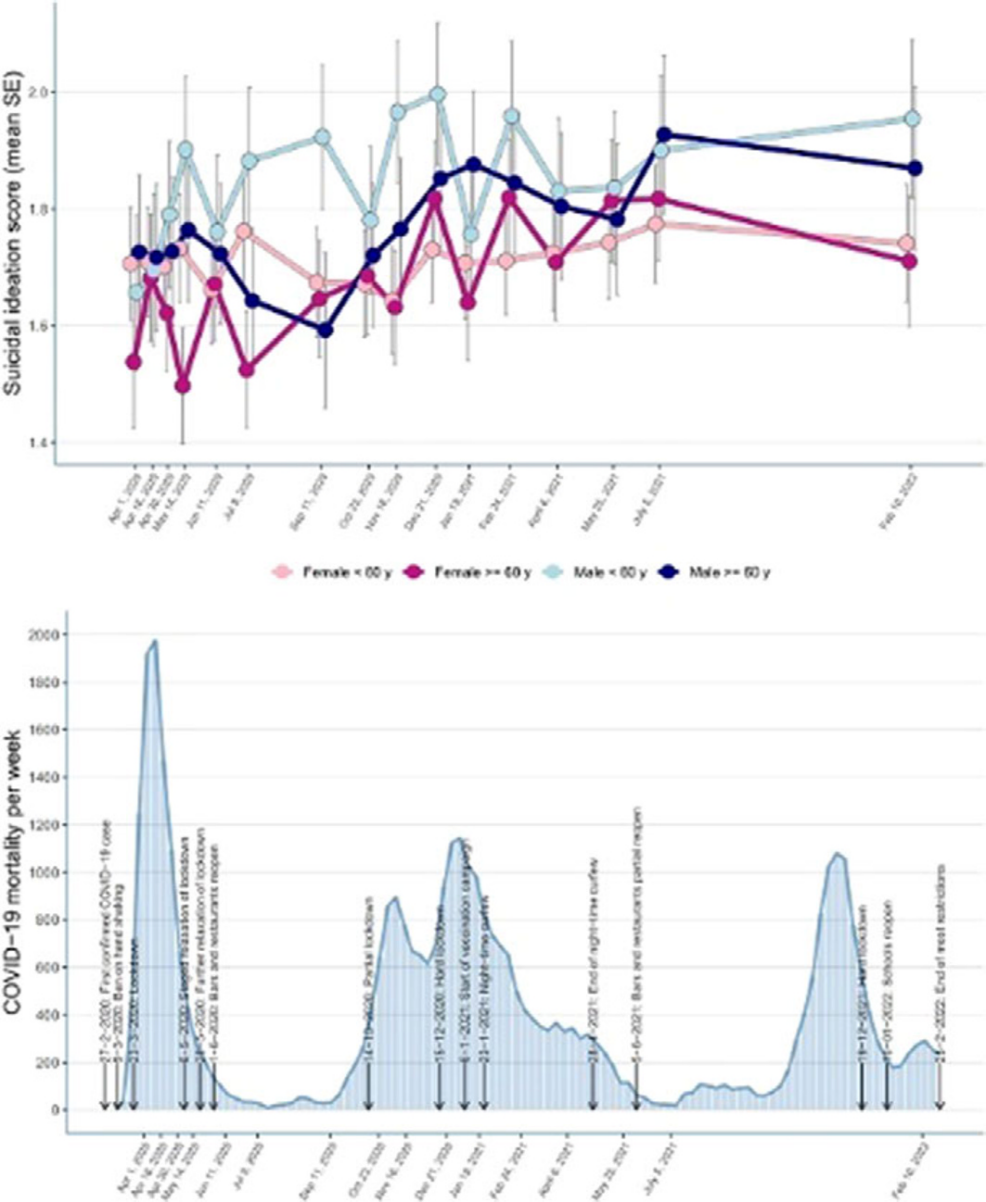
Image 1: Long description.

**Image 2:**
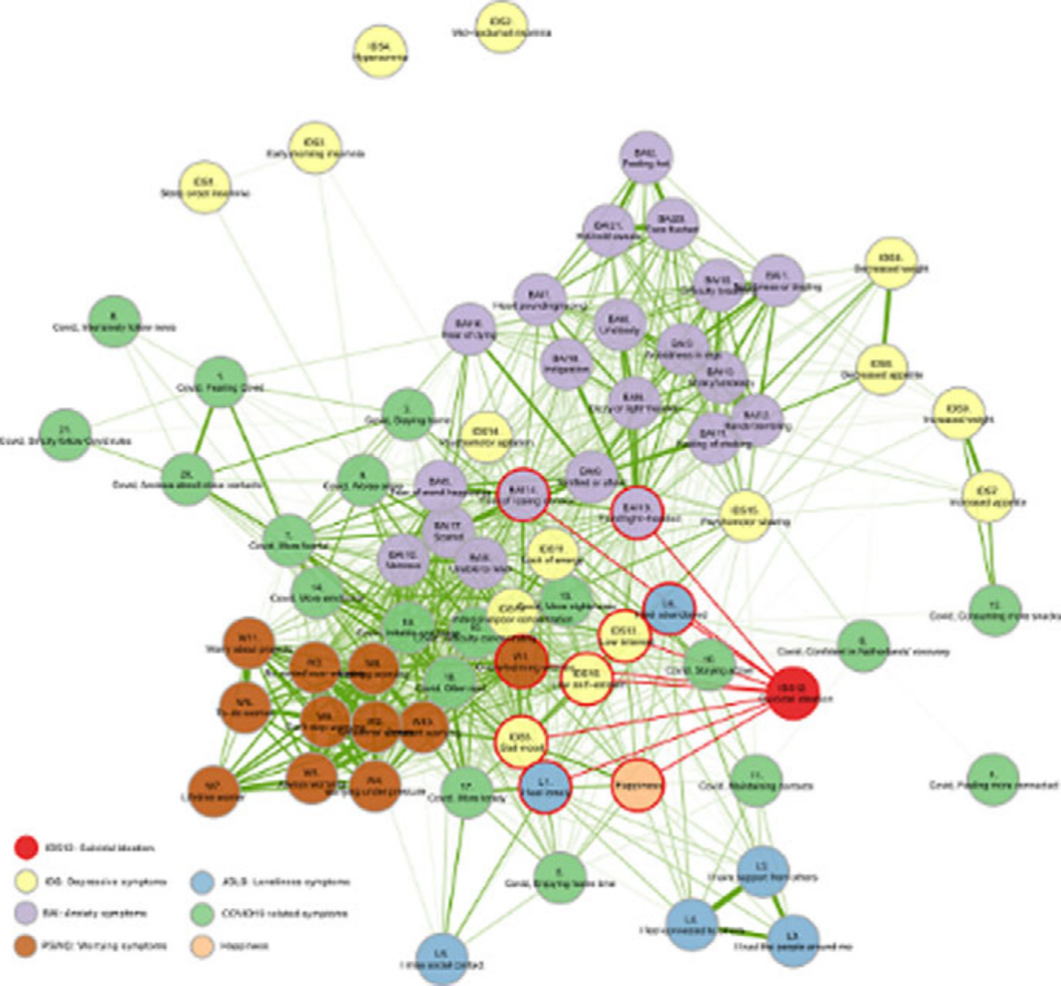
Image 2: Long description.

**Image 3:**
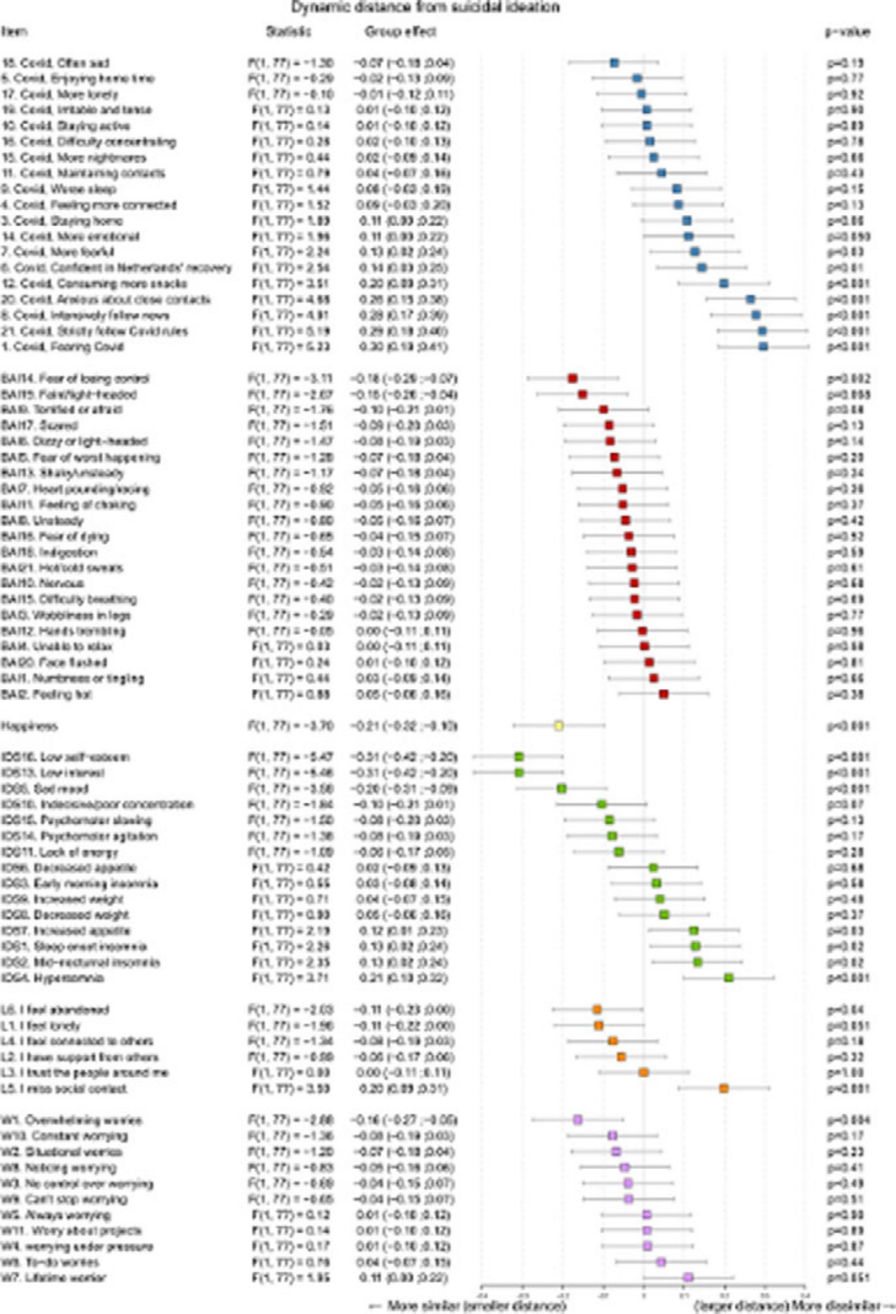
Image 3: Long description.

**Conclusions:**

SI co-fluctuated with emotional, anxiety, social and worry related symptoms supporting the view that suicidality is embedded in broader networks of emotional and cognitive symptoms. These findings highlight the value of personalized, time-sensitive monitoring approaches that can help detect critical periods of increased suicide risk and may guide more targeted interventions.

**Disclosure of Interest:**

None Declared

## Long descriptions

### Image 1: Long description

The top panel is a line graph showing Suicidal ideation score mean S E on the Y axis ranging from 1.4 to 2.0. The X axis shows dates from April 1 2020 to February 10 2022. Four lines represent demographics. Male less than 60 y light blue generally shows the highest scores, peaking near 2.0 in late 2020. Male greater than or equal to 60 y dark blue and Female greater than or equal to 60 y magenta show fluctuating trends that converge upward over time. Female less than 60 y light pink remains the lowest or second lowest throughout. All groups show a sharp increase in ideation scores during the initial lockdown period.

The bottom panel is an area chart showing C O V I D 19 mortality per week on the Y axis from 0 to 2000. The X axis aligns with the top panel. The data shows three distinct peaks. The first peak in early 2020 reaches nearly 2000 deaths. The second peak in late 2020 reaches approximately 1100 deaths. The third peak in early 2022 reaches approximately 1100 deaths. Vertical arrows with text labels mark specific events including.

* 27-2-2020 First confirmed C O V I D 19 case.

* 21-3-2020 Lockdown.

* 11-5-2020 Staged relaxation of lockdown.

* 14-10-2020 Partial lockdown.

* 15-12-2020 Hard lockdown.

* 6-1-2021 Start of vaccination campaign.

* 5-6-2021 Bars and restaurants partial reopen.

* 19-12-2021 Hard lockdown.

* 25-2-2022 End of most restrictions.

Navigate back to Image 1:.

### Image 2: Long description

A network diagram with nodes of varying sizes and colors connected by green and red lines. A legend at the bottom left identifies the categories.

* Red node: I D S 12 Suicidal ideation, located on the right side with thick red connections to central nodes.

* Yellow nodes (I D S Depressive symptoms): Distributed across the top and right, including I D S 4 Hypersomnia, I D S 1 Early morning insomnia, and I D S 8 Decreased appetite.

* Purple nodes (B A I Anxiety symptoms): Concentrated in the upper-center, including B A I 4 Fear of dying, B A I 16 Fear of losing control, and B A I 1 Numbness or tingling.

* Orange nodes (P S W Q Worrying symptoms): Clustered in the lower-left, including W 11 Worry about projects and W 1 Constant worrying.

* Green nodes (C O V I D 19 related symptoms): Spread throughout the left and bottom, such as node 1 Covid Feeling bored and node 12 Covid Consuming more snacks.

* Blue nodes (A S L S Loneliness symptoms): Located at the bottom right, including L 5 I feel support from others and L 4 I feel connected to others.

* Peach node: Happiness, located at the bottom center.

Thick green lines indicate strong positive correlations, while red lines indicate negative correlations or specific pathways to suicidal ideation. The central hub is densely packed with B A I and I D S nodes, showing high connectivity between anxiety and depression symptoms.

Navigate back to Image 2:.

### Image 3: Long description

The forest plot is organized into five color-coded sections. Each row contains an item name, a Statistic F(1, 77), a Group effect value with confidence intervals, a visual plot of the effect, and a p-value.

* The first section (blue squares) contains 18 Covid-related items. Items like ‘Fearing Covid’ and ‘Strictly follow Covid rules’ show the largest positive group effects (0.30 and 0.29 respectively) with p-values of 0.001, indicating they are more dissimilar to suicidal ideation.

* The second section (red squares) lists 22 B A I items related to anxiety. ‘Fear of losing control’ shows the most negative group effect (-0.18, p = 0.002), while most other items cluster near zero with p-values above 0.05.

* The third section (green squares) includes ‘Happiness’ and 15 I D S items. ‘Happiness’ has a significant negative effect (-0.21, p = 0.001). ‘Low self-esteem’ and ‘Low interest’ show the strongest negative correlations (-0.31, p < 0.001), while ‘Hypersomnia’ shows a positive effect (0.21, p = 0.007).

* The fourth section (orange squares) lists 6 L S items. ‘I feel abandoned’ and ‘I feel lonely’ show negative effects (-0.11), while ‘I miss social contact’ shows a positive effect (0.20, p = 0.001).

* The fifth section (purple squares) lists 11 W items related to worry. ‘Overwhelming worries’ has the strongest negative effect (-0.16, p = 0.004), while ‘Lifetime worrier’ has a positive effect (0.11, p = 0.051).

The x-axis at the bottom is labeled ‘More similar (smaller distance)' on the left and ‘More dissimilar (larger distance)' on the right, centered around a zero-effect vertical dashed line.

Navigate back to Image 3:.

